# Establishment and characterization of a highly metastatic hepatocellular carcinoma cell line

**DOI:** 10.1080/21655979.2023.2296775

**Published:** 2024-01-07

**Authors:** Jiacheng Huang, Mengqing Sun, Menglan Wang, Anning Yu, Huilin Zheng, Chiwen Bu, Jie Zhou, Yu Zhang, Yiting Qiao, Zhenhua Hu

**Affiliations:** a Department of Hepatobiliary and Pancreatic Surgery, The Fourth Affiliated Hospital, International Institutes of Medicine, Zhejiang University School of Medicine, Yiwu, China; b Jinan Microecological Biomedicine Shandong Laboratory, Jinan, China; c Division of Hepatobiliary and Pancreatic Surgery, Department of Surgery, The First Affiliated Hospital, Zhejiang University School of Medicine, Hangzhou, China; d School of Pharmacy and Department of Hepatology, the Affiliated Hospital of Hangzhou Normal University, Hangzhou Normal University, Hangzhou, China; e School of Medicine, Zhejiang University, Hangzhou, China; f School of Biological and Chemical Engineering, Zhejiang University of Science and Technology, Hangzhou, China; g Department of General Surgery, People’s Hospital of Guanyun County, Lianyungang, China

**Keywords:** Hepatocellular carcinoma, cell line, xenograft, primary culture, metastasis

## Abstract

The prevalence of alcohol-related hepatocellular carcinoma (HCC) has been increasing during the last decade. Cancer research requires cell lines suitable for both *in vitro* and *in vivo* assays. However, there is a lack of cell lines with a high *in vivo* metastatic capacity for this HCC subtype. Herein, a new HCC cell line was established, named HCC-ZJ, using cells from a patient diagnosed with alcohol-related HCC. The karyotype of HCC-ZJ was 46, XY, del (p11.2). Whole-exome sequencing identified several genetic variations in HCC-Z that occur frequently in alcohol-associated HCC, such as mutations in *TERT*, *CTNNB1*, *ARID1A*, *CDKN2A*, *SMARCA2*, and *HGF*. Cell counting kit-8 assays, colony formation assays, and Transwell assays were performed to evaluate the proliferation, migration, and sensitivity to sorafenib and lenvatinib of HCC-Z *in vitro*. HCC-ZJ showed a robust proliferation rate, a weak foci-forming ability, a strong migration capacity, and a moderate invasion tendency *in vitro*. Finally, the tumorigenicity and metastatic capacity of HCC-Z were evaluated using a subcutaneous xenograft model, an orthotopic xenograft model, and a tail-veil injection model. HCCZJ exhibited strong tumorigenicity in the subcutaneous xenograft and orthotopic tumor models. Moreover, HCC-ZJ spontaneously formed pulmonary metastases in the orthotopic tumor model. In summary, a new HCC cell line derived from a patient with alcohol-related HCC was established, which showed a high metastatic capacity and could be applied for *in vitro* and *in vivo* experiments during pre-clinical research.

Highlights

• An alcohol-related HCC cell line, HCC-ZJ, was established

• HCC-ZJ was applicable for *in vitro* functional experiment and gene editing

• HCC-ZJ was applicable for *in vivo* tumor growth and spontaneous metastasis models

## Introduction

Hepatocellular carcinoma (HCC) is one of the most fatal diseases worldwide, with a mortality ranking third among cancer-related deaths [1]. HCC is the most commonly diagnosed type of liver cancer (approximately 75%). The incidences of HCC in Asia and Africa are ranked first in the world, and China has the largest HCC population (approximately 1.4 billion) [2]. Most patients with HCC are diagnosed at 75 years old or younger, and the incidence rate in males is two to four fold higher than that in females [3]. Metastasis is one of the major factors that lead to treatment failure and death of patients, and the metastatic rate of HCC is over 50%, even in small HCC [4]. The etiology of HCC is multifactorial, but has not yet been fully determined. Common risk factors include hepatitis B virus (HBV) or hepatitis C virus infection, alcohol consumption, and aflatoxin-contaminated food [5], all of which contribute to chronic liver inflammation. Progress has been made in field of HCC therapeutic strategies, such as surgical treatment, chemotherapy, radiofrequency ablation, targeted therapy, immunotherapy, and nanoparticle synthesis in drug delivery [6–10]. With the increasing popularity of hepatitis B vaccination, the rise in the rate of HBV-associated HCC has started to decline. However, the incidence of alcohol-related HCC showed rapid growth during the last decade [11], causing great concern among oncologists. Previous studies have demonstrated alcohol consumption as a risk factor for HCC. A case-control study showed that a 10-year history of daily alcohol consumption over 80 grams was associated with a 5-fold increase in the incidence of HCC in an Italian population [12]. Another retrospective study of an American cohort also identified alcohol as an independent risk factor, and subjects consuming more than 1500 grams of alcohol per year were significantly more susceptible to HCC [13]. The mechanisms by which alcohol increases HCC incidence include, but are not limited to, genotoxicity, aberrant metabolism, dysregulated epigenetic processes, and impaired immune surveillance [14]. However, there is still a lack of feasible therapeutic targets for alcohol-associated HCC. Considering its increasing incidence and mortality, *in vitro* and *in vivo* models for this subtype of HCC are urgently needed for basic and pharmaceutical research, and in general, the complexity of HCC subtypes requires the development of further HCC models.

Currently, there are two major types of HCC model for medical research, namely *in vitro* and *in vivo* models. HCC cell lines are the most common HCC models for *in vitro* studies, with advantages of convenient large-scale preparation, high accessibility for genetic engineering, and affordable experiment costs. In-depth molecular biology studies or highthroughput screening in cell lines *in vitro* often identify critical oncogenes and tumor suppressors, whose potential as therapeutic targets could be further verified using *in vivo* models, such as transgenic animal models and patientderived xenografts. Cell lines often preserve features of their original lesions, and there is a high level of heterogeneity in biological behavior and experimental applications among various HCC cell lines. For example, MHCC97H and MHCC97L cell lines were derived from the metastatic lesion and primary tumor of the same 39 years-old Chinese male patient with HCC with HBV infection, among which MHCC97H shows a significantly higher trend of forming pulmonary metastatic lesions in nude mice compared with MHCC97L. Another well-known HCC cell line, Huh7, which was established from a 57 year old Japanese male, is highly susceptible to HCV infection, thus making it an ideal *in vitro* model to investigate the molecular mechanisms and therapeutic targets for HCV infection [15].

Subcutaneous and orthotopic inoculation of HCC cell lines is routinely applied to construct *in vivo* models of HCC, bridging the gap between petri dishes and complicated physiological conditions encountered during pre-clinical research. Although subcutaneous xenografts lack a liver-specific microenvironment, they are still highly useful because of the integration of vesiculation, stroma, immune cells, and acellular components, and are convenient for continuous measurement of tumor growth using vernier calipers. Orthotopic inoculation of HCC cell lines in hepatic lobes can mostly reproduce the tumor microenvironment, blood circulation, and lymph circulation, making it an ideal model to study not only primary HCC, but also distal metastasis. Tian et al. (1999) established MHCC97H, which was tumorigenic and lungmetastatic in nude mice [16]. Yan et al. (2013) established a tumorigenic and lymph node metastatic HCC cell line by transplanting fresh human surgical specimens into nonobese diabetic/severe combined immunodeficiency (NOD/SCID) mice [17]. In addition, The PLC/PRF/5 cell line could also form tumors in the liver of nude mice. However, very few HCC cell lines can spontaneously metastasize to the lung at a high efficiency. Therefore, the limited choice of cell lines for *in vivo* models, especially orthotopic HCC models with a metastatic capacity, increases the chance of biased observation during research. Therefore, there is an urgent need to establish more HCC cell lines to increase the diversity of experimental models, especially those capable of forming orthotopic tumors and spontaneous pulmonary metastases.

In the present study, an HCC cell line was established from a patient with alcoholassociated HCC, which showed strong tumorigenicity and metastasis *in vivo*. HCC-ZJ was demonstrated as suitable for long-term passage and cell function analysis using standard tissue culture procedures *in vitro*. Karyotyping and whole exome sequencing (WES) were performed to identify potential genetic variations. In addition, its tumorigenicity was evaluated in both subcutaneous and orthotopic models, and its tendency form pulmonary metastasis was analyzed in the orthotopic model and a tail-vein injection model.

## Materials and methods

### Reagents

The following reagents were used in this study: William’s E medium (Cat# PM151213, Procell, Wuhan, China); Fetal Bovine Serum (FBS) (Cat# 04–002-1A, Biological Industries, Haemek, Israel); Dulbecco’s modified Eagle’s medium (DMEM) (Cat# 06-1055-57-1A, Biological Industries); Antibiotic-Antimycotic solution (Cat# P7630, Solarbio, Beijing, China); Recombinant Human Hepatocyte Growth Factor (HGF) (Cat# 100–39, PeproTech, Cranbury, NJ, USA); Recombinant Human Insulin (Cat# I8830, Solarbio); Dexamethasone Sodium Phosphate (Cat# D6950, Solarbio); Rat tail tendon collagen type I (Cat# C8062, Solarbio); Deoxyribonuclease I (Cat# D8071, Solarbio); Collagenase type IV (Cat# MB-125-0050, Rockland Immunochemicals Inc., Pottstown, PA, USA); Penicillin-Streptomycin (10×) Solution (Cat# 03–031, Biological Industries); Roswell Park Memorial Institute 1640 medium (RPMI1640) (Cat# 01–100-1A, Biological Industries); Cell Counting Kit-8 (CCK-8) (Cat# HY-K0301, MedChemExpress, Monmouth Junction, NJ, USA); Matrigel Matrix (Cat# 356255, Corning Inc., Corning, NY, USA); D-Luciferin potassium salt (Cat# ST196, Beyotime, Jiangsu, China); Rabbit monoclonal antibody recognizing Cytokeratin 7 (Cat# ab181598, Abcam, Cambridge, UK); Cytokeratin 19 polyclonal antibodies (Cat# 10712–1-AP, Proteintech, Rosemont, IL, USA); Sorafenib (Cat# S7397, Selleck, Houston, TX, USA); Lenvatinib (Cat# HY-10981, MedChemExpress); 4% Paraformaldehyde Fix Solution (Cat# P0099, Beyotime); PolyJet™ In Vitro DNA Transfection Reagent (Cat# SL100688, SignaGen, Frederick, MD, USA); and Blasticidin S (Cat# B9300, Solarbio).

### Specimen source

This study was approved by the Clinical Research Ethics Committee of the First Affiliated Hospital, Zhejiang University School of Medicine (Reference number: IIT20200623A). A 53-year-old Chinese male was admitted to the Division of Hepatobiliary and Pancreatic Surgery, Department of surgery, First Affiliated Hospital, Zhejiang University School of Medicine (Hangzhou, China) because of elevated serum alpha fetal protein (AFP). Informed consent was acquired from the participant in this study. Radiotherapy, chemotherapy, and radiofrequency ablation were not performed preoperatively.

### Primary culture of HCC-ZJ

Complete William’s E medium comprised William’s E medium, 10% FBS, 1% antibiotic-antimycotic solution, recombinant human HGF (5 ng/mL), recombinant human insulin (0.5 U/mL), and dexamethasone sodium phosphate (1.6 μmol/L). The digestive buffer comprised complete William’s E medium, deoxyribonuclease I (0.02 mg/mL), and collagenase type IV (2 mg/mL).

Fresh tumor tissue was obtained from the patient, stored in chilled complete William’s E medium, and transferred to laboratory within 30 min. Tumor tissue was dissected into pieces and then incubated with digestive buffer in a cell incubator (humidified, 5% CO_2_) for 3 h. The digested mixture was filtered through a 70 μm strainer, the filtrate was centrifuged at 1200 rpm for 5 min, and the supernatant was discarded. After washing with phosphate-buffered saline (PBS) three times, the pellet was resuspended in complete William’s E medium. Finally, the cell resuspension solution was seeded into 6-well plates coated with rat tail tendon collagen following the manufacturer’s instructions. The complete William’s E medium was changed every 3 days. When the confluence of HCC-ZJ reached 80–90%, the cells were passaged at a ratio of 1:5. As the passage number increased, the percentage of fibroblasts and macrophages gradually decreased. After being passaged for more than 30 generations, the cultured cells were cryopreserved in cell freezing medium containing 70% DMEM, 20% FBS, and 10% dimethyl sulfoxide (DMSO). The cell line was named HCC-ZJ (Zhejiang), and was tested to be mycoplasma-free using a MycoBlue™ Mycoplasma Detector (Cat# D101–01, Vazyme, Nanjing, China). When the passage number reached 100, the culture medium was changed to DMEM supplemented with 10% FBS and penicillinstreptomycin solution.

### Identification of short tandem repeats

To rule out the possibility that the cultured HCC-ZJ was contaminated by known cell lines, patterns of short tandem repeats (STRs) of HCC-ZJ, the corresponding whole blood sample, and DNA extracted from a paraffin-embedded tumor sample were analyzed. The STR information of HCC-ZJ, the blood, and DNA extracted from paraffin-embedded tumor sample was cross-referenced to 2455 known cell lines recorded in the ATCC, DSMZ, JCRB, and RIKEN databases by applying DSMZ tools. The tumor DNA was extracted from the paraffin-embedded tumor sample using a QIAamp DNA FFPE Tissue Kit (Cat# 56404, Qiagen, Hilden, Germany).

### Cell proliferation properties

HCC cell lines HCCLM3 (maintained in RPMI-1640 medium), Huh1 (maintained in DMEM medium), and HCC-ZJ (maintained in DMEM medium) were seeded in 96 wells plates at a density of 2000 cells/well or 4000 cells/well (four replicates were performed each day). A CCK-8 assay was then applied according to the manufacturer’s instructions. In addition, the sensitivity of HCCLM3, Huh1, and HCC-ZJ to sorafenib and lenvatinib was also assessed using CCK-8 assays (five replicates were performed for each concentration).

### Colony formation assay

HCCLM3, Huh1, and HCC-ZJ cells were seeded in 6-wells plates at a density of 2000 cells/well or 4000 cells/well. After incubation in a 5% CO_2_ incubator at 37°C for 14 days, the formed cell colonies were fixed using 4% paraformaldehyde for 15 minutes, and stained with 5% crystal violet for 15 minutes. Three replicates were performed.

### Transwell assays

Transwell assays to evaluate cell migration and invasion were performed using 24-well Transwell inserts (Cat# 353504, Falcon, Brookings, SD, USA) sealed using an 8 μm pore polycarbonate membrane (Cat# 353097, Falcon). Transwell inner chambers coated with 50 μL of diluted Matrigel (volume of original Matrigel:FBS-free DMEM or RPMI1640 = 1:8) were used for the invasion assay, and these chambers were incubated at 37°C for one hour until the gel was set before use. Uncoated chambers were applied for the migration assay. 100 μL of FBS-free DMEM or RPMI1640 with 10^5^ cells was added to the inner chamber, and 600 μL of DMEM or RPMI1640 with 20% FBS was added to the outside wells. After incubation for 24 h for the migration assay, or 48 h for the invasion assay, the cells inside the chambers were wiped away. The migrated cells on the bottom of the chambers were fixed using 4% paraformaldehyde and stained with Crystal violet. The cells were then counted under an optical microscope. Three replicates were performed.

### Lentivirus infection

Plasmid pLenti-CMV-V5-LUC-Blast (w567–1) was chosen as the vector to introduce the luciferase gene into HCC-ZJ. Briefly, a mixture of the plasmid and PolyJet™ In Vitro DNA Transfection Reagent was added to the medium of HEK293T cells at approximately 60% confluence. After 8 h, the medium was changed for fresh complete medium. After 48 h, the medium containing the lentivirus was collected and filtered through 0.45-μm filters. Then, HCC-ZJ cells were treated with the lentivirus-containing medium for 12 h, after which the medium was replaced with fresh complete medium and incubation continued for another 12 h. The infection procedure was repeated three times, and infected HCC-ZJ (hereafter referred to as HCC-ZJ-Luc) were selected against Blasticidin S (5 μg/mL) in complete medium for one week. Both fluorescence signal detection and quantitative real-time reverse transcription PCR (qRT-PCR) of the luciferase gene were performed to evaluate the infection efficiency.

### Subcutaneous and orthotopic tumorigenicity in BALB/C nude mice

All animal experiments were approved by the Animal Experiment Ethical Inspection of the First Affiliated Hospital, Zhejiang University School of Medicine (Reference number: 2021907). Our animal study adhered to the Animal Research: Reporting of *In Vivo* Experiments (ARRIVE) guidelines. BALB/C nude mice were obtained from Hangzhou Ziyuan Laboratory Animal Science and Technology Co. Ltd. (Hangzhou, China). All mice were kept in cages under Specific Pathogen Free conditions, and five or six mice were allocated into each group in one cage in the present study.

HCC-ZJ-Luc was resuspended at a density of 2 × 10^7^ cells/mL. The cell suspension was mixed with an equal volume of Matrigel matrix, and 0.1 mL of the mixture was injected subcutaneously into the left flanks of 6-week old male BALB/C nude mice (approximate 10^6^ cells per mouse). The mice were checked every 3 days, and then sacrificed humanely once the diameter of tumor reached 2 cm. The subcutaneous tumor samples were dissected, and fixed using 10% formalin. The volume of the subcutaneous tumor was calculated using equation (*):

$$V=A\times B^{2}÷2\left(*\right)$$

V represents the volume of the tumor; A represents the maximum diameter of the tumor; and B represents the minimum diameter of the tumor.

HCC-ZJ-Luc and HCCLM3-Luc cells were resuspended at a density of 2 × 10^8^ cells/mL. The cell suspension was mixed with an equal volume of Matrigel matrix. BALB/C nude mice were anesthetized by intraperitoneal injection of 0.3% pentobarbital (0.1 mL/10 g). Then, an incision was made approximately 1 cm under the xiphoid of the mice using scissors, and the left lobe of the liver was gently squeezed out. Next, 10 μL of the mixture was injected into the left lobe of liver (approximate 10^6^ cells per mouse). After the Matrigel mix was set, the left lobe was placed into the abdomen, and the abdominal incision was sutured. D-Luciferin potassium salt was injected intraperitoneally to visualize the location and size of tumors 1 week after orthotopic implantation, and the signals were imaged using IVIS Spectrum In Vivo Imaging System (Perkin-Elmer, Waltham, MA, USA). Imaging was performed once a week, and the mice were sacrificed at the end of the third imaging cycle. The lungs were dissected and imaged 10 mins after the injection of D-Luciferin potassium salt, and the fluorescent signals in the lung were measured. Orthotopic tumors and lungs were fixed using 10% formalin, stained with hematoxylin-eosin (H&E), and examined to validate the existence of metastasis loci in the lung.

### Immunohistochemistry

To validate whether the HCC-ZJ cell line was hepatocellular carcinoma or intrahepatic cholangiocarcinoma, we performed immunohistochemistry (IHC) for cytokeratin 7 (CK7) and cytokeratin 19 (CK19) in the orthotopic tumors. The detailed process of IHC has been described previously [18,19]. The positive fraction of CK7 and CK19 were then automatic analyzed using the IHC Profiler [20] plugin in ImageJ (NIH, Bethesda, MD, USA), and three random fields were analyzed.

### Whole-exome sequencing

QIAamp Fast DNA tissue kit (Cat# 51404, Qiagen) was applied to extract the genomic DNA from HCC-ZJ cells. The DNA was quantified using a Nanodrop spectrophotometer (Thermo Fisher Scientific, Waltham, MA, USA), and 1% agarose electrophoresis was applied to assess the integrity of the DNA sample. The sequencing library was constructed using a SureSelect Human All Exon V6 kit (Agilent Technologies, Santa Clara, CA, USA), and sequenced using an Illumina Nova-seq6000 instrument (Illumina Inc., San Diego, CA, USA).

The raw data were in the fastq format, and were pre-processed to eliminate adaptors, low quality reads, and short reads (less than 75 bp) using Fastp (version 0.20.0) [21]. FastQC (version 0.11.8), Multiqc (version 1.7), and Qualimap (version 2.2.1) were applied to evaluate the quality of both the raw data and the clean data. The clean data were aligned to the human reference genome (GRCh37) using BWA (version 0.7.17) [22]. After being sorted and indexed using SAMtools (version 1.9) [23], bam files were processed using the GATK pipeline (version 4.1.9.0) [24], including recalibration, marking of PCR duplicates, and variation calling. Single nucleotide polymorphisms (SNPs) and insertion/deletions (INDELs) were identified. Multiple annotation databases, including Refseq, 1000 Genomes, the Exome Aggregation Consortium (EXAC), esp6500, SIFT, clinvar, PolyPhen, MutationTaster, COSMIC, gwasCatalog, and OMIM, were used as references for SNP and INDEL annotation when using ANNOVAR [25]. In addition, copy number variation (CNV) was identified using CNVkit (version 0.9.8) [26], and structural variations (SVs) were inferred using Lumpy software (version 0.3.1) [27]. All the sequencing and bioinformatic analyses were carried out by OE Biotech Co. Ltd. (Shanghai, China).

### Statistical analysis

All experiments were biologically replicated at least three times. Continuous variables are presented as mean ± standard deviation (SD). The number of mice in each group was not precalculated. A two-tailed Student’s T test was applied to compare the positive fraction of CK7 and CK19. *P* < 0.05 was considered statistically significant. GraphPad prism was applied for the statistical analysis.

## Results

### Patients’ hospital course

The patient was negative for hepatitis virus from type A to type E and human immunodeficiency virus (HIV). *Clonorchis sinensis* and *Treponema pallidum* were also excluded in this case. The detailed preoperative laboratory examination results are listed in Table 1. D-dimmer was less than 170 μg/L (ref: 0–700 μg/L). Serum prostate specific antigen (PSA), alpha fetoprotein (AFP), protein-II induced by vitamin K absence (PIVKA-II), and ferritin were 9.325 ng/mL (ref: 0.000–4.000), 368.9 ng/mL (ref: 0.0–20.0), 2036 mAU/mL (ref: 0–40), and 536.5 ng/mL (ref: 7.0–323.0), respectively. The international normalized ratio (INR) and prothrombin time (PT) were 1.25 (ref: 0.85–1.15) and 14.3 s (ref: 10.0–13.5), respectively. Serum total protein, albumin, and globulin were 50.3 g/L (ref: 65.0–85.0), 33.8 g/L (ref: 40.0–55.0), and 16.5 g/L (ref: 20.0–40.0), respectively. Alanine aminotransaminase (ALT) and aspartate aminotransferase (AST) were 914 U/L (ref: 9–50) and 1404 U/L (ref: 15–40). Total cholesterol, high-density lipoprotein (HDL), and low-density lipoprotein (LDL) were 1.91 mmol/L (ref: 3.14–5.86), 0.59 mmol/L (ref: 0.78–1.81), and 0.86 mmol/L (ref: 1.31–3.29), respectively. Abdominal ultrasonography and computed tomography (CT) and magnetic resonance imaging (MRI) revealed a 4.5 cm diameter mass occupying in S8 of the fatty and cirrhotic liver (Supplementary Figure S1, Supplementary Figure S2A). The patients stated that he had a habit of drinking approximately 150 ml of liquor every day for 20 years.

**Table 1.** Preoperative laboratory examination of the patients.

Tests	Results	Units	Reference Range
D-dimmer	170	μg/L	0–700 μg/L
Serum prostate specific antigen (PSA)	9.325	ng/ml	0.000–4.000 ng/ml
Alpha fetoprotein (AFP)	368.9	ng/ml	0.0–20.0 ng/ml
Protein-II induced by vitamin K absence (PIVKA-II)	2036	mAU/ml	0–40 mAU/ml
Ferritin	536.5	ng/ml	7.0–323.0 ng/ml
International normalized ratio (INR)	1.25		0.85–1.15
Prothrombin time (PT)	14.3	s	10.0–13.5 s
Serum total protein	50.3	g/L	65.0–85.0 g/L
Serum albumin	33.8	g/L	40.0–55.0 g/L
Serum globulin	16.5	g/L	20.0–40.0 g/L
Alanine aminotransaminase (ALT)	914	U/L	9–50 U/L
Aspartate aminotransferase (AST)	1404	U/L	15–40 U/L
Total cholesterol	1.91	mmol/L	3.14–5.86 mmol/L
High-density lipoprotein (HDL)	0.59	mmol/L	0.78–1.81 mmol/L
Low-density lipoprotein (LDL)	0.86	mmol/L	1.31–3.29 mmol/L

Partial hepatectomy was performed after routine preoperative examinations. Two pathologists independently performed pathological examinations according to Guidelines for the diagnosis and treatment of hepatocellular carcinoma (2019 edition) [28]. Postoperative pathological examination confirmed the mass was composed of tumor cells derived from hepatocytes and biliary epithelial cells. Postoperative immunohistochemistry revealed that AFP and glypican-3 (GPC-3) were negative, and HepPar-1, CD34, CD117, CK7, CK19, MUC-1, Arginase-1, and Gelsolin were partially positive (Supplementary Figure S2B-K).

### Identification of HCC-ZJ and its karyotype

**Figure 1.**
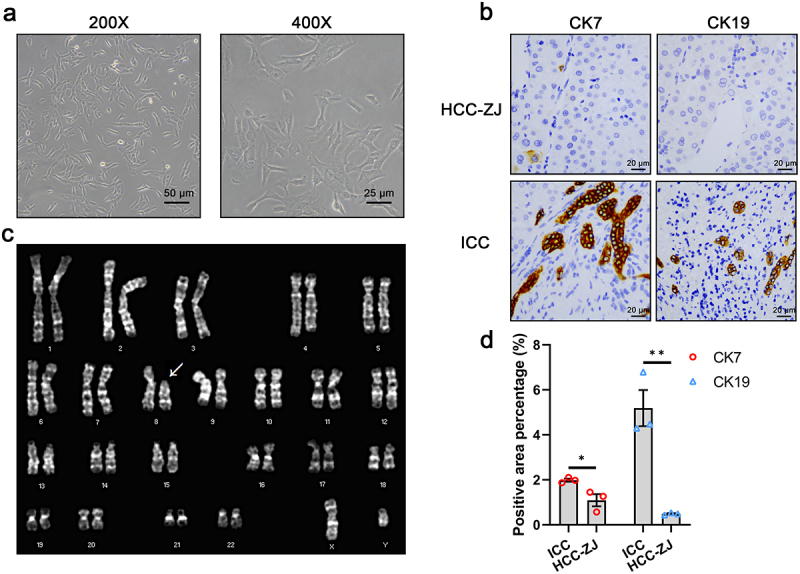
The morphology of HCC-ZJ cells (a); immunohistochemistry for CK7 and CK19 (b); the karyotype of HCC-ZJ cells (c); the CK7 and CK19 positive rate of HCC-ZJ and intrahepatic cholangiocarcinoma (ICC) samples (*n* = 3) (d). * represents *P* < 0.05 and ** represents *P* < 0.01.

The morphology of HCC-ZJ cells under the light microscope is shown in Figure 1(a). STR analysis was conducted to verify the source of this cell line and exclude the contamination of other cell lines in the laboratory. The detailed STR information for HCC-ZJ, the patient’s peripheral blood, and DNA extracted from the paraffinembedded tumor sample is listed in Table 2. The results were consistent among the samples except for Allele 1 of microsatellite D21S11. Moreover, the chance of contamination from other cell lines with known STR fingerprints was excluded for HCC-ZJ. The IHC results for CK7 and CK19 revealed that HCC-ZJ was not derived from biliary epithelial cells Figure 1(b-d).

**Table 2.** Short tandem repeat (STR) identification of HCC-ZJ.

Microsatellite (chromosome)	HCC-ZJ		Patients’ blood		DNA from FFPE tissue	
	Allele1	Allele2	Allele1	Allele2	Allele1	Allele2
D5S818	10	11	10	11	10	11
D13S317	9	11	9	11	9	11
D7S820	9	11	9	11	9	11
D16S539	9	10	9	10	9	10
VWA	17	17	17	17	17	17
TH01	9	9	9	9	9	9
AMEL	X	Y	X	Y	X	Y
TPOX	8	8	8	8	8	8
CSF1PO	12	13	12	13	12	13
D12S391	17	19	17	19	17	19
FGA	19	24	19	24	19	24
D2S1338	17	19	17	19	17	19
D21S11	31	31	30	31	30	31
D18S51	14	22	14	22	14	22
D8S1179	14	14	14	14	14	14
D3S1358	15	15	15	15	15	15
D6S1043	14	19	14	19	14	19
PENTAE	5	12	5	12	5	12
D19S433	13	14	13	14	13	14
PENTAD	9	9	9	9	9	9
D1S1656	13	15	13	15	13	15

The karyotyping result identified a breakage at the short arm of chromosome 8, zone 11, band 2 in HCC-ZJ; however, the region from the break point to the end of the short arm was missing. No other heteroploidy, polyploidy, or chromosomal translocation was observed. Therefore, the representative karyotype of HCC-ZJ was described as 46, XY, del [8](p11.2) Figure 1(c).

### Genetic variation analysis of the HCC-ZJ cell line

According to an exome sequencing study in a cohort containing 87 pairs of HCC and normal tissues by Cleary (2013), pivotal genes including *TP53*, *CTNNB1*, *KEAP1*, *C16orf62*, *MLL4*, and *RAC2* exhibited recurrent mutations [29]. In a larger cohort consisting of 243 patients with HCC, *TP53*, *CTNNB1*, *AXIN1*, *ALB*, *ARID1A*, *ARID2*, *ACVR2A*, *NFE2L2*, *RPS6KA3*, *KEAP1*, *RPL22*, *CDKN2A*, *CDKN1A*, and *RB1* were identified as driver genes for HCC [30]. Moreover, the authors of the latter study observed that the exposure to different risk factors would lead to divergent genetic variation patterns. For example, mutations of *TERT* (60%), *CTNNB1* (37%), *ARID1A* (13%), *CDKN2A* (9%), *SMARCA2* (3%), and *HGF* (3%) were significantly associated with the history of alcohol abuse, while the mutation of *TP53* and *IL6ST* frequently occurred in HCC patients with HBV history [30].

**Figure 2.**
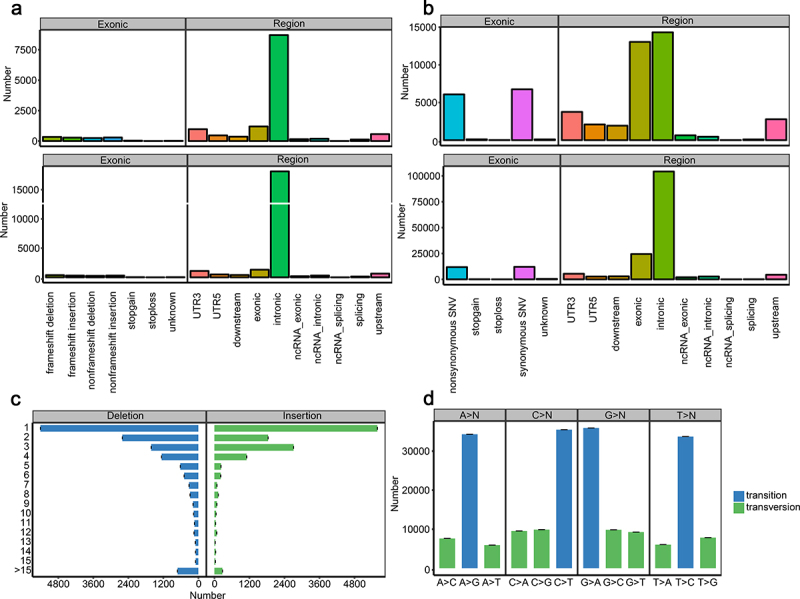
Statistics of the whole-exome sequencing. (a) the numbers and mutation types of genes (upper) and sites (lower) from INDELs; (b) the numbers and mutation types of genes (upper) and sites (lower) from SNPs; (c) the numbers of INDEL sites containing the corresponding number of bases; (d) the number of transitions and transversions based on the four kinds of bases.

**Figure 3.**
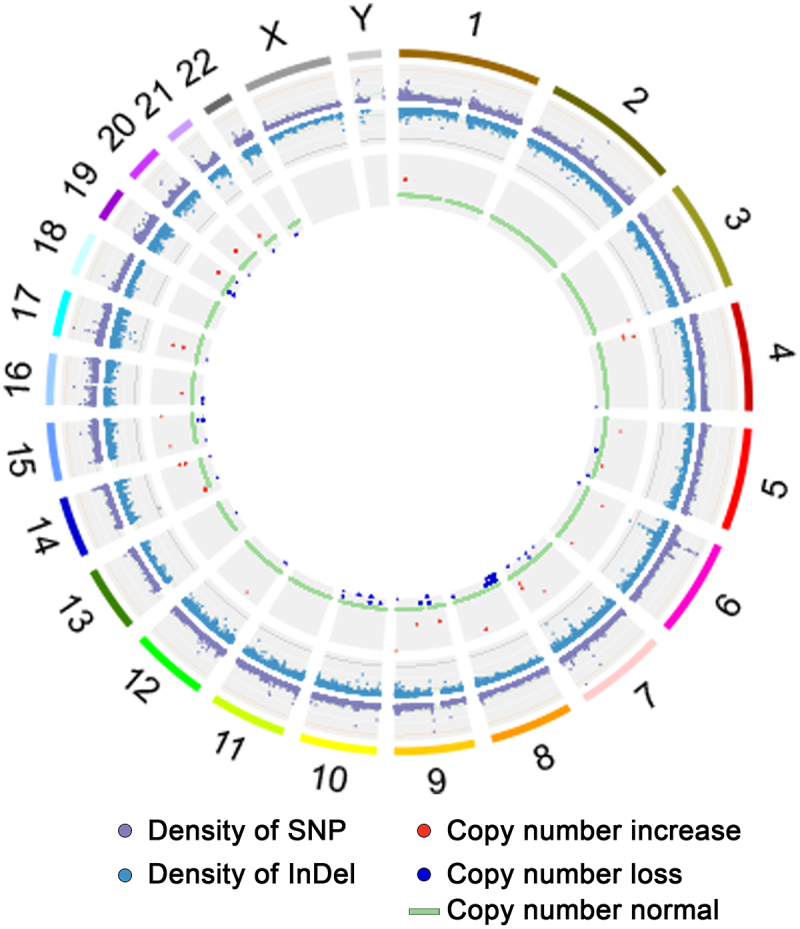
Circos plot describing the distribution of SNPs, INDELs, copy number variants (CNVs), and structural variations (SVs) in genome.

To analyze whether the genetic variations of HCC-ZJ could partially reflect the longterm alcohol exposure history of the patient who provided the specimen, the genetic variations of HCC-ZJ were analyzed using WES. In total 205,943 SNP sites and 26,915 INDEL sites were identified in 39,082 and 12,723 genes, respectively. The landscape of the exome sequencing of HCC-ZJ is displayed in Figures 2 and 3. We retrieved the data of aforementioned frequently mutated genes in HCC-ZJ, and observed several nonsynonymous single nucleotide variant (SNV) sites in *TP53*, *CTNNB1*, *ARID1A*, *NFE2L2*, and *CDKN1A* (Table 3), as well as several frameshift INDELs in *TERT*, *ARID1A*, *CDKN2A*, *SMARCA2*, *HGF*, *TP53*, *RAC2*, *ALB*, *ARID1A*, *ARID2*, *ACVR2A*, and *RB1* (Table 4). In general, the mutation landscape of HCC-ZJ exhibited similar characteristics to that of reported alcohol-associated HCC.

**Table 3.** Nonsynonymous single nucleotide variant (SNV) of HCC-ZJ cell by whole-exome sequencing.

Chromosome	Position	Reference nucleotide	Variant nucleotide	Gene	Region annotation	Variant type	Genotype (GT)	Allele depth (AD)	Q value
17	7579472	G	C	TP53	exonic	nonsynonymous SNV	0/1	31,29	720.64
3	41268766	A	C	CTNNB1	exonic	nonsynonymous SNV	0/1	22,21	415.64
1	27056319	C	T	ARID1A	exonic	stopgain	0/1	15,12	297.64
2	178095983	A	T	NFE2L2	exonic	nonsynonymous SNV	0/1	76,60	1490.64
2	178098948	T	G	NFE2L2	exonic	nonsynonymous SNV	0/1	8,9	259.64
2	178098965	T	G	NFE2L2	exonic	nonsynonymous SNV	0/1	7,9	278.64
6	36645696	A	G	CDKN1A	exonic	nonsynonymous SNV	1/1	0,123	3750.06
6	36652339	C	G	CDKN1A	exonic	nonsynonymous SNV	1/1	1,65	2106.06

**Table 4.** Frameshift insertion/deletion (INDEL) of HCC-ZJ cell by whole-exome sequencing.

Chromosome	Position	Reference nucleotide	Variant nucleotide	Gene	Region annotation	Variant type	Genotype (GT)	Allele depth (AD)	Q value
5	1302113	A	AC	TERT	Intergenic	Frameshift insertion	1/1	0,4	128.1
1	27099687	GC	G	ARID1A	Intronic	Frameshift deletion	0/1	1,2	71.6
1	27105819	CA	C	ARID1A	Exonic	Frameshift deletion	0/1	37,46	1447.6
1	27107263	T	TC	ARID1A	3’ UTR	Frameshift insertion	0/1	4,3	56.6
9	21968446	TCCAGGCGCGCCGACCGCTCAAGCGC TCCAGGTCCACCCGGCGGAGGGCAG AGAAAGCGCGACCGCGCGGCCCGCA GGGTTGCAAGAAGAAAACGAGTGTT ATATAATGAGTCTCAGTGGTTGCTCACAATG	T	CDKN2A	3’ UTR	Frameshift deletion	0/1	1,2	51.6
9	1899810	GTT	G	SMARCA2	Intergenic	Frameshift deletion	1/1	0,1	35.44
9	2058479	GC	G	SMARCA2	Intronic	Frameshift deletion	0/1	4,2	64.6
9	2058481	AG	A	SMARCA2	Intronic	Frameshift deletion	0/1	4,2	64.6
9	2097548	GA	G	SMARCA2	Intronic	Frameshift deletion	1/1	0,8	210
9	2133646	TA	T	SMARCA2	Intronic	Frameshift deletion	1/1	0,1	35.44
9	2149334	A	AT	SMARCA2	Intronic	Frameshift insertion	1/1	0,1	35.44
9	2160736	TGA	T	SMARCA2	Intronic	Frameshift deletion	1/1	0,10	412.02
9	2207472	ATTTT	A	SMARCA2	Intergenic	Frameshift deletion	1/1	0,1	35.44
9	2598723	GAA	G	SMARCA2	Intergenic	Frameshift deletion	1/1	0,2	64.28
7	81359116	CA	C	HGF	Intronic	Frameshift deletion	1/1	0,13	370.02
17	7577678	CT	C	TP53	Intronic	Frameshift deletion	0/1	6,11	212.6
17	7578711	CTTTT	C	TP53	Intronic	Frameshift deletion	1/1	0,1	35.44
17	7579643	CCCCCAGCCCTCCAGGT	C	TP53	Intronic	Frameshift deletion	0/1	5,31	1237.6
22	37622881	TGG	T	RAC2	Intronic	Frameshift deletion	0/1	30,17	607.6
22	37628053	A	AG	RAC2	Intronic	Frameshift insertion	0/1	32,24	652.6
4	74282092	A	AT	ALB	Intronic	Frameshift insertion	1/1	0,4	102.1
1	27099687	GC	G	ARID1A	Intronic	Frameshift deletion	0/1	1,2	71.6
1	27105819	CA	C	ARID1A	Exonic	Frameshift deletion	0/1	37,46	1447.6
1	27107263	T	TC	ARID1A	3’ UTR	Frameshift insertion	0/1	4,3	56.6
12	45879268	AG	A	ARID2	Intergenic	Frameshift deletion	0/1	2,3	84.6
2	148293282	CTTCCTTCCTTTCTTCCTTTCTTTCTTCTTCT	C	ACVR2A	Intergenic	Frameshift deletion	1/1	0,1	35.44
2	148320287	CT	C	ACVR2A	Intergenic	Frameshift deletion	1/1	0,2	49.28
2	148680896	TGGAC	T	ACVR2A	Intronic	Frameshift deletion	1/1	0,2	78.28
13	48953655	C	CA	RB1	Intronic	Frameshift insertion	0/1	1,6	120.73
13	48955659	C	CAA	RB1	Intronic	Frameshift insertion	0/1	1,2	73.6

### In vitro *biological behaviors of HCC-ZJ*

**Figure 4.**
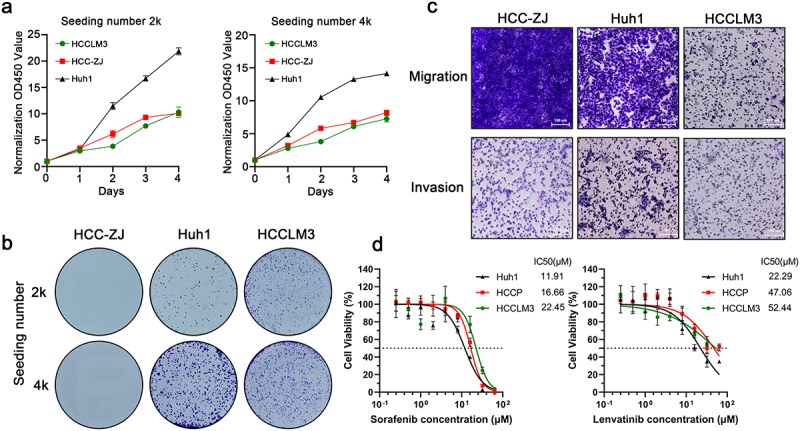
*In vitro* characteristics of HCC-ZJ cells. (a) the proliferative potential of HCC-ZJ, Huh1, and HCCLM3 cells (*n* = 4); (b) colony formation ability of HCC-ZJ, Huh1, and HCCLM3 cells (*n* = 3); (c) migration and invasion abilities of HCC-ZJ, Huh1, and HCCLM3 cells (*n* = 3);(d) the sensitivity of HCC-ZJ, Huh1, and HCCLM3 to sorafenib (left) and lenvatinib (right) (*n* = 5).

The *in vitro* biological behaviors of HCC-ZJ were analyzed using routine cell function experiments, including proliferation assays, 2D colony formation assays, Transwell assays, and drug toxicity assays. In the proliferation assays, the doubling times of HCCZJ, HCCLM3, and Huh1 were 27 h, 28 h, and 21 h, respectively Figure 4(a). In the 2D colony formation assays, HCC-ZJ formed significantly fewer colonies than HCCLM3 and Huh1 Figure 4(b), suggesting that parental HCC-ZJ was not suitable for this assay in the absence of treatments that enhanced tumorigenicity. In the Transwell migration assays without the coating of thick extracellular matrix, the migratory capacity of HCC-ZJ was significantly stronger than that of HCCLM3 and Huh1 Figure 4(c). However, in the Transwell invasion assay with the thick Matrigel coating, the invasive capacity of HCC-ZJ was similar to that of HCCLM3, but slightly weaker compared with that of Huh1 Figure 4(c). In the drug toxicity assay, the half maximal inhibitory concentration (IC50) values of HCCLM3, HCC-ZJ, and Huh1 to sorafenib were 22.45 μM, 16.66 μM, and 11.91 μM, respectively, while their IC50 values to lenvatinib were 52.44 μM, 47.06 μM, and 22.29 μM, respectively Figure 4(d).

In general, HCC-ZJ was suitable for routine *in vitro* experiments including proliferation assays, Transwell assays, and drug toxicity assays.

### Subcutaneous and orthotopic tumorigenicity of HCC-ZJ

To investigate whether HCC-ZJ could be applied as an HCC model *in vivo*, subcutaneous xenograft, orthotopic tumor, and spontaneous pulmonary metastasis models were established.

**Figure 5.**
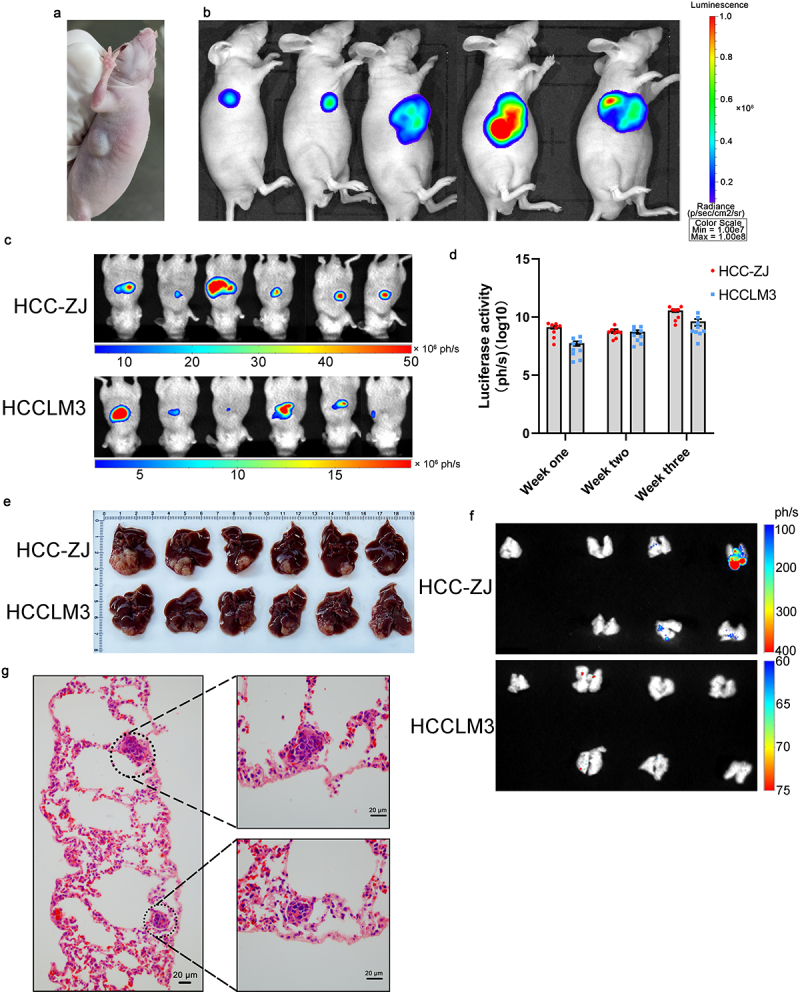
*In vivo* oncogenicity of the HCC-ZJ cell line. (a) subcutaneous inoculation of 1 × 10^6^ HCC-ZJ cells; (b) subcutaneous inoculation of 1 × 10^6^ HCC-ZJ-Luc cells; the luminescence reached 1 × 10^8^ (*n* = 5); (c) orthotopic tumorigenesis of 1 × 10^6^ HCC-ZJ-Luc and HCCLM3-Luc cells (*n* = 6) in the third week; (d) luciferase activity of HCC-ZJ-Luc and HCCLM3-Luc at the indicated time point; (e) dissection of orthotopic tumors (*n* = 6) in the third week; (f) imaging of newly dissected lungs from HCC-ZJ-bearing and HCCLM3-bearing mice showed that orthotopic tumors of HCC-ZJ could migrate into the lung (*n* = 6); the first lung of each group derived from healthy mice without orthotopic inoculation of HCC cells; (g) H&E staining demonstrating the lung metastasis of orthotopic HCC-ZJ.

Five nude mice underwent subcutaneous inoculation of 10^6^ HCC-ZJ cells expressing luciferase (HCC-ZJ-Luc). Thirty days later, all mice developed subcutaneous tumors with fluorescent signals in the presence of the luciferin substrate Figure 5(a,b). The average volume of the tumors was 1101.7 mm^3^.

To analyze HCC-ZJ’s capacity to form orthotopic tumors and spontaneous pulmonary metastasis, six nude mice underwent orthotopic inoculation of HCC-ZJ-Luc and HCCLM3-Luc. Fluorescent signals could be detected after one week, which steadily increased and reached 5 × 10^7^ (ph/s) at the end of the third week Figure 5(c), indicating that HCC-ZJ could form tumors rapidly in the liver lobe microenvironment Figure 5(c-e). The orthotopic tumor of HCCLM3 grew slower than HCC-ZJ Figure 5(c-e). Immediately after the mice were sacrificed, livers and lungs were dissected and imaged, and fluorescent signals could be detected in four lungs among all the lung samples Figure 5(f). However, the fluorescent signals of lung metastasis were detected only in two mice inoculated with HCCLM3, and the signal intensity was weaker Figure 5(f). H&E staining also demonstrated the existence of multiple micro metastatic lesions in the lungs of orthotopic HCC models established using HCC-ZJ Figure 5(g). However, in another type of spontaneous pulmonary metastasis model constructed by tail-vein injection of HCC cells, metastatic foci could not be detected in mice that received HCCZJ-Luc according fluorescent imaging (Supplementary Figure S3).

In general, HCC-ZJ could be applied for subcutaneous xenograft, orthotopic liver tumors, and spontaneous pulmonary metastasis models *in vivo*.

## Discussion

In the present work, an HCC cell line was established from a patient with a long history of excessive alcohol consumption and named HCC-ZJ. This cell line is sustainable in routine tissue culture for long-term passage. Its proliferation rate in the petri dish was similar to that of HCCLM3, but slower compared with that of Huh1. The invasiveness of HCC-ZJ was similar to that of HCCLM3 and Huh1, while its migratory capacity was strongest among these cell lines. The sensitivity of HCC-ZJ to sorafenib and lenvatinib lies between that of HCCLM3 and Huh1. Most importantly, HCC-ZJ can form both ectopic and orthotropic tumors in nude mice, and spontaneous pulmonary metastasis could be achieved in the orthotopic HCC model.

HCC-ZJ exhibits the loss of a long segment in the short arm of chromosome 8, and a similar genomic aberration has also been reported by other karyotyping studies of HCC tissues. For example, Devi et al. (2010) observed chromosomal aberrations in 1q, 5p, 8p, 16q, and 17p in patients with HCC with alcohol addiction, hepatitis infection, and hemochromatosis [31]. Emi et al. (1992) calculated the frequency of heterozygous loss of chromosome 8p in different cancer types in their cohort, finding that HCC possessed the highest frequency of allele loss of 8p [32]. In addition, several other researchers also reported the loss of heterozygosity of chromosome 8p in HCC [33–35]. Moreover, the deletion of chromosome 8p correlates with poor differentiation [36,37], advanced stage [36], large tumor size [37], metastasis [38], and adverse prognosis [39]. These findings suggest that some critical tumor suppressors for HCC might be localized in this region. Indeed, several genes within chromosome 8p have been identified to suppress the malignant transformation, proliferation, and metastasis of HCC cells, including *DLC1* [40], *MSRA* [41], and *HTPAP* [42]. Thus, HCC-ZJ might be a good cell model to study the correlation between aberrant chromosome 8p and HCC progression.

We identified multiple genetic variations in genes with known association with longterm alcohol consumption in HCC-ZJ, including *TERT*, *CTNNB1*, *ARID1A*, *CDKN2A*, and *SMARCA2* [30,43]. The protein product of TERT is a telomerase reverse transcriptase, which maintains the length of telomere ends by adding the telomere repeat TTAGGG, and its mutation has been detected in approximate 85% of human cancers [30,44–46]. A meta-analysis covering 1939 patients discovered that the mutation of *TERT* occurred more frequently in patients with non-viral HCC than in HCC associated with hepatitis virus infection [47]. The protein encoded by *CTNNB1* is β-catenin, a component of constitute adherent junctions (AJs), which plays pivotal roles in the Wnt/β-catenin pathway. In patients with HCC with different etiologies, such as HBV, HCV, and alcohol, mutations of *CTNNB1* are often localized in exon 3, which encodes a protein domain with multiple phosphorylation sites involved in its continuous activation [30,48], and a similar pattern was observed in HCC-ZJ. *ARID1A* and *SMARCA2* participate in the process of chromatin remodeling. Two independent cohorts have demonstrated the significant correlation between *ARID1A* mutation and patients with non-viral HCC, especially subjects with high alcohol consumption [30,49]; however, the correlation between *SMARCA2* and alcohol-associated HCC requires further validation. The protein product of *CDKN2A* impedes the degradation of tumor suppressor protein p53 by interacting with its E3 ubiquitin-protein ligase MDM2, and mutation of *CDKN2A* can lead to aberrant cell cycle progression in multiple types of cancer [50]. In general, HCC-ZJ shows genetic variations in these critical genes associated with the malignant progression of HCC, especially alcohol-associated HCC, making it a useful tool for the development of targeted therapies.

Currently, HCC cell lines, such as HCCLM3, Hep3B, HepG2, and Huh1, are widely applied in mechanistic and translational research; however, there remains an urgent need for more HCC cell lines because the application of many conventional cell lines is limited. For example, some HCC cell lines widely used for *in vitro* experiments, such as HepG2 and HLE, are not highly tumorigenic in nude mice. Similarly, some cell lines, such as Mahlavu, with a potent migratory tendency *in vitro*, cannot form spontaneous pulmonary metastasis *in vivo*. Sometimes, scientists modify the commercially available HCC cells or establish novel HCC cells lines to cater for different scientific purposes. For instance, HCCLM3 cells, with a high capacity for pulmonary metastasis, were isolated from the pulmonary metastatic lesion in nude mice after MHCC97H cells were injected subcutaneously for 3 cycles [51]. Recently, more HCC cell lines with various features have been established via primary culture of HCC tissues. For example, Lee et al. (1999) established and characterized four primary HCC cell lines with HBV DNA [52]. Cheung et al. (2014) established and characterized a primary HCC cell line that possessed a high metastasis ability [53]. Herein, an HCC cell line from a patient with long-term alcohol consumption was established, which might represent a practical tool to study alcoholassociated HCC both *in vitro* and *in vivo*.

Several published works aimed to augment the HCC cell line bank. Lou et al. (2004) established the FHCC-98 line, which was tumorigenic in nude mice; however, its metastatic capacity was not determined [54]. Park et al. (1995) characterized eight HCC cell lines from Korean patients [55]; however, only the *in vitro* characteristics of these cell lines were evaluated. Additionally, classical HCC cell lines MHCC97H and HCCLM3 were established previously [16,56], both of which easily metastasize and are HBV positive. The HCC-ZJ cell established in the present study enlarged the bank of HCC cell lines that are characterized by a high metastatic capacity. Furthermore, until now, no alcoholrelated HCC cell line had been established.

Several peculiarities of HCC-ZJ were observed in the *in vitro* and *in vivo* experiments. Firstly, HCC-ZJ seldom formed colonies when cultured under a low cell density. The most reasonable explanation was that the human HCC tissue-derived cells needed to overcome the adherence and proliferative stress during the transformation from the original liver microenvironment to a plastic dish where adherence molecules were lost. Additionally, under low cell density, the weak cell-cell contact threatened the survival of the cells. Secondly, pulmonary metastasis was not observed in the tail vein model, while it was detected in the orthotopic model. There are three possible explanations for this phenomenon. First, Matrigel was applied for the orthotopic implantation of HCCZJ, which greatly promoted the colonization of tumor cells. Second, the liver microenvironment could form a favorable niche for tumor growth and the migration of orthotopic tumor cells in the vasculature were encompassed and sheltered by clots and macrophages [57]. Third, the tumor cells were confronted with immune cells without sanctuary in the tail vein model, and were easily obliterated by the immune system.

## Conclusion

The present work established a new HCC cell line derived from alcohol-associated HCC, named HCC-ZJ. HCC-ZJ was shown to be useful to carry out proliferation and metastasis assays *in vitro*, and it is applicable for *in vivo* tumor growth and spontaneous metastasis models. The HCC-ZJ cell line has several potential future applications: as a promising model for alcohol-related HCC; in a variety of *in vitro* experiments, such as gene editing, evaluation of proliferation, migration, and invasion abilities; as an ideal *in vivo* model to evaluate spontaneous pulmonary metastasis; and could be the object for drug screening under the two- or three-dimensional cultivation based on organoids, biological microarrays, and histologically engineered livers.

## Supplementary Material

Supplemental Material

## Data Availability

The data presented in this study are available on request from the corresponding author. The data are not publicly available due to the patient’s privacy.
