# Supplementary figures and images for: Establishment and characterization of a highly metastatic hepatocellular carcinoma cell line
Source: Bioengineered. 2024 Jan 7;15(1):2296775. doi: 10.1080/21655979.2023.2296775 (PMC10773622; doi:10.1080/21655979.2023.2296775)

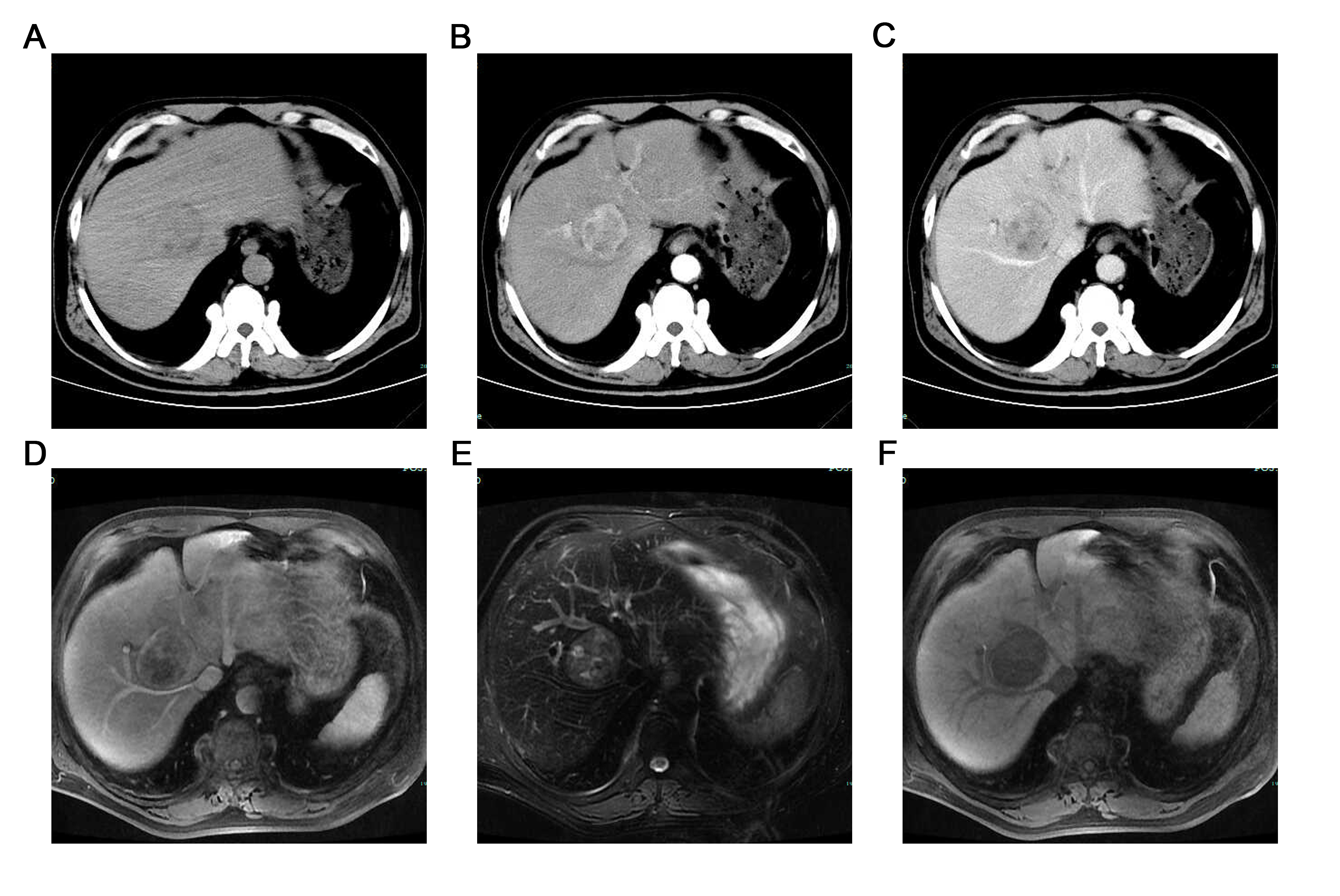

Supplement: Supplemental Material [file KBIE_A_2296775_SM4593.zip › SFigure1.tif]

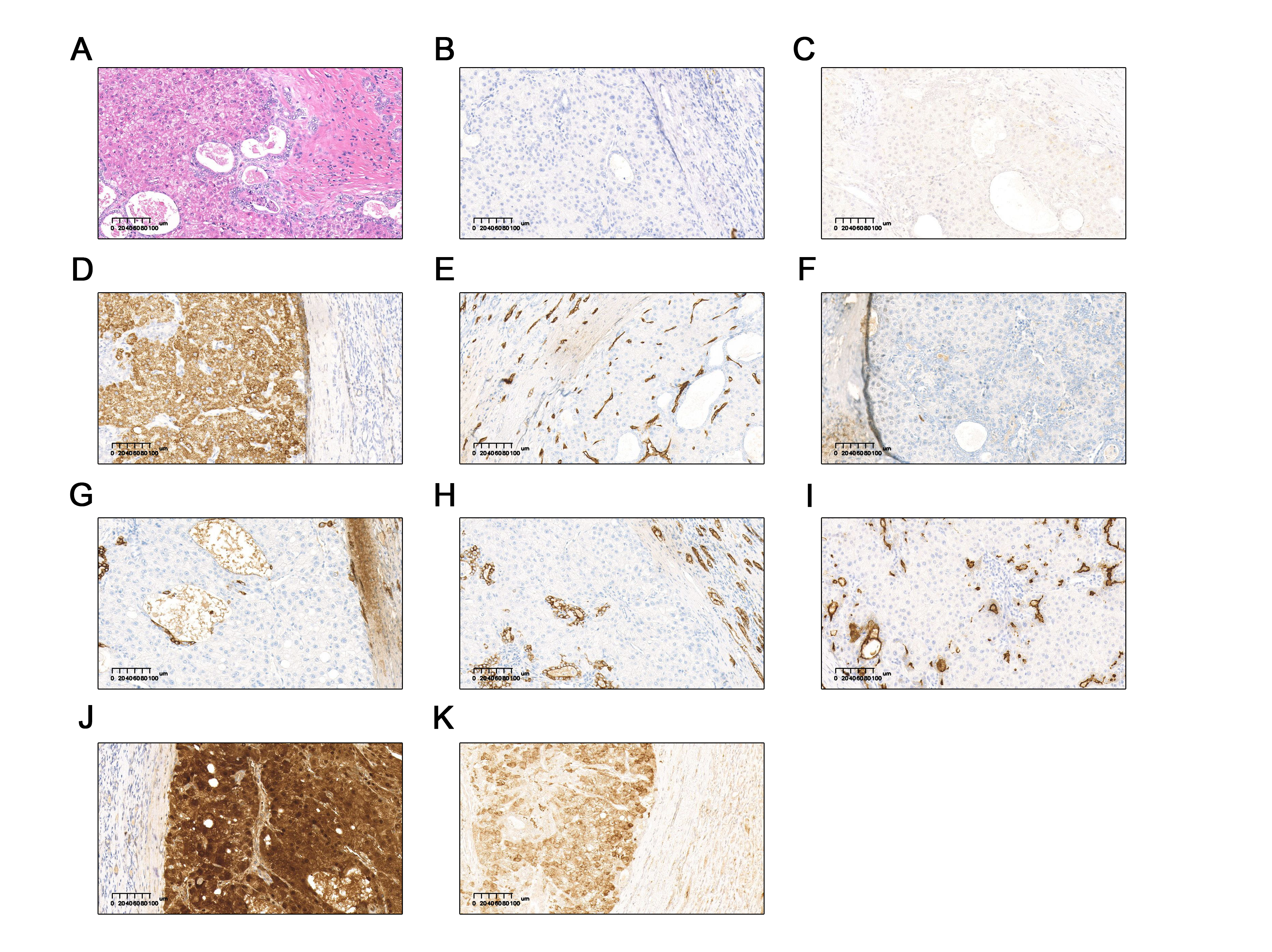

Supplement: Supplemental Material [file KBIE_A_2296775_SM4593.zip › SFigure2.tif]

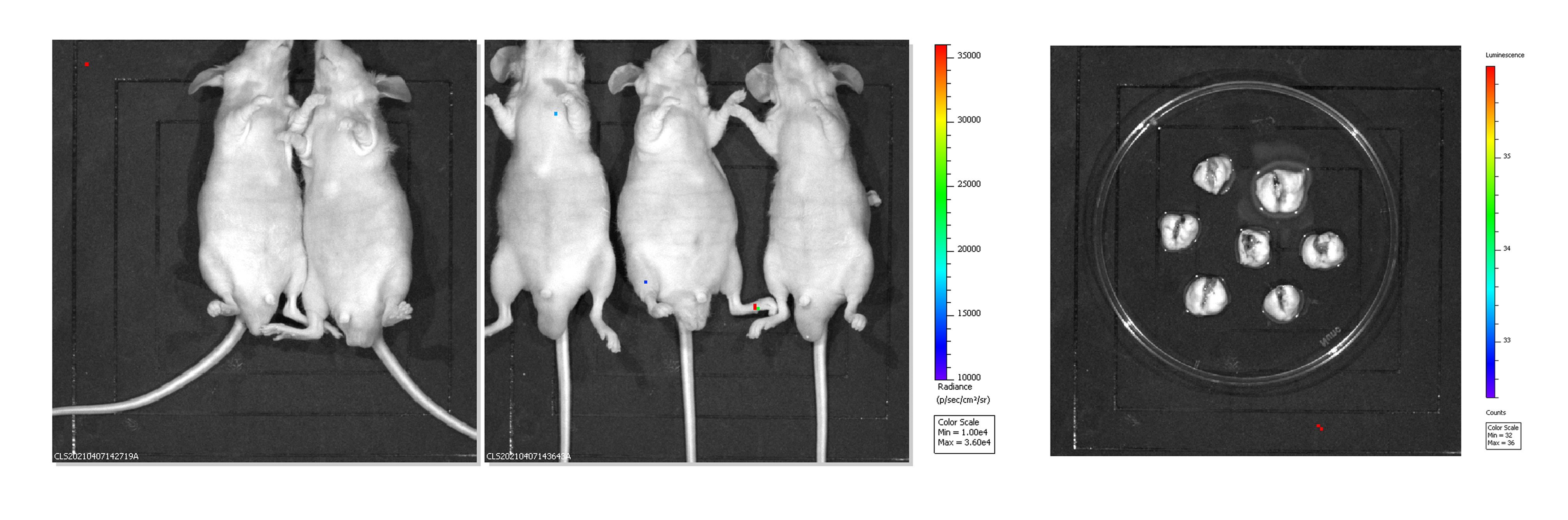

Supplement: Supplemental Material [file KBIE_A_2296775_SM4593.zip › SFigure3.tif]
